# Field-cycling NMR with high-resolution detection under magic-angle spinning: determination of field-window for nuclear hyperpolarization in a photosynthetic reaction center

**DOI:** 10.1038/s41598-017-10413-y

**Published:** 2017-09-21

**Authors:** Daniel Gräsing, Pavlo Bielytskyi, Isaac F. Céspedes-Camacho, A. Alia, Thorsten Marquardsen, Frank Engelke, Jörg Matysik

**Affiliations:** 1 Institut für Analytische Chemie, Universität Leipzig, Linnéstraße 3, D-04103 Leipzig, Germany; 2 Escuela de Química, Tecnológico de Costa Rica, Sede Central, 30101 Cartago, Costa Rica; 3 Institut für Medizinische Physik und Biophysik, Universität Leipzig, Härtelstr. 16-18, D-04107 Leipzig, Germany; 4 Leiden Institute of Chemistry, 2333, Leiden, The Netherlands; 5 Bruker BioSpin GmbH, Silberstreifen 4, D-76287 Rheinstetten, Germany

## Abstract

Several parameters in NMR depend on the magnetic field strength. Field-cycling NMR is an elegant way to explore the field dependence of these properties. The technique is well developed for solution state and in relaxometry. Here, a shuttle system with magic-angle spinning (MAS) detection is presented to allow for field-dependent studies on solids. The function of this system is demonstrated by exploring the magnetic field dependence of the solid-state photochemically induced nuclear polarization (photo-CIDNP) effect. The effect allows for strong nuclear spin-hyperpolarization in light-induced spin-correlated radical pairs (SCRPs) under solid-state conditions. To this end, ^13^C MAS NMR is applied to a photosynthetic reaction center (RC) of the purple bacterium *Rhodobacter (R.) sphaeroides* wildtype (WT). For induction of the effect in the stray field of the magnet and its subsequent observation at 9.4 T under MAS NMR conditions, the sample is shuttled by the use of an aerodynamically driven sample transfer technique. In the RC, we observe the effect down to 0.25 T allowing to determine the window for the occurrence of the effect to be between about 0.2 and 20 T.

## Introduction

To study magnetic-field dependent properties as, for example, the relaxation times *T*_1_ and *T*_2_^1–5^ as well as the production of nuclear spin-hyperpolarization by different mechanisms^1,6,7^ depending on field matching, various field-cycling systems have been realized in NMR methodology. These field-cycling systems induce spin dynamics or spin hyperpolarization at a low magnetic field and measure NMR at high magnetic field. For liquid-state NMR, either samples^3,8–12^ or NMR probes^1^ can be shuttled between the evolution/polarization field and the measurement field. Solids are shuttled in relaxation experiments which do not require chemical shift resolution^2,4,5,13^ or the sample is not moved between fields but the magnetic field is provided by an electro-magnet with rapidly switched electric current^2^. Here we present a device that combines aerodynamic field-cycling with high-resolution detection under magic-angle spinning (MAS) conditions.

To test the function of the new setup, we explore the magnetic field window for the occurrence of the solid-state photo-CIDNP (photochemically induced dynamic nuclear polarization) effect. This phenomenon is caused by the spin-dynamics of a spin-correlated radical pair (SCRP) under solid-state conditions^14–16^. In solid-state NMR, the effect induces high nuclear spin-hyperpolarization simply by illumination with visible light. Therefore, it opens an appealing trajectory to improve selectivity and sensitivity in NMR spectroscopy and imaging^15,17,18^. Furthermore, photo-CIDNP MAS NMR provides insights on the electronic structures of both the electronic ground-state and the charge-separated state allowing to study electron transfer on the atomic scale.

In systems showing the solid-state photo-CIDNP effect, the SCRP is created upon photo-excitation of the primary electron donor which leads to a charge-separated state via an electron transport to an acceptor molecule (Fig. S1). The SCRP evolves under the difference in electron Zeeman frequencies, the nuclear Zeeman frequency, the secular as well as the pseudo-secular part of the electron-nuclear hyperfine interaction (hfi) and the electron-electron dipolar interaction^15,16^. This spin-evolution leads to singlet-to-triplet inter-conversion and to the creation of nuclear hyperpolarization. Two mechanisms have been proposed to produce photo-CIDNP in WT RCs under solid-state conditions^15^: The three-spin mixing (TSM) mechanism^15,17^ occurring upon interconversion under influence of the pseudo-secular part of the hyperfine interaction and coupling between the two electron spins, causes hyperpolarization which appears as emissive (negative) signals in the high-field ^13^C MAS NMR spectra^6,19^. Additionally, the differential decay (DD) mechanism occurs due to the pseudo-secular hyperfine coupling and different decay rates of the radical-pair singlet state to the electronic ground state and the radical-pair triplet state to the molecular donor triplet state, causing absorptive signals in the high-field ^13^C MAS NMR spectra^18^. The sign of the polarization caused by the DD is enhanced-absorptive (positive) but the TSM is expected to overrule the DD^6,19^. According to the theory, there are two windows for the solid-state photo-CIDNP effect, one around earth’ magnetic field (ca. 50 μT), the other reaching to the range of high-field NMR spectrometers^15,16,20^.

It remains puzzling that the solid-state photo-CIDNP effect has been observed in *all* natural photosynthetic reaction centers (RCs) which were studied^14,21–25^ and in some flavin proteins^7,26^, but in no other electron-transfer systems. Since the theory predicts strong magnetic field dependence, and since photo-CIDNP MAS NMR is mainly done at 4.7 T (200 MHz ^1^H frequency) and 9.4 T (400 MHz), it simply might be that NMR experiments do not match with the magnetic field conditions required. Hence, to explore new and non-photosynthetic electron transfer systems, more fields need to be available.

Since the photosynthetic primary reaction is highly efficient, the solid-state photo-CIDNP effect has been proposed to be conserved in evolution and to correlate with functional relevance^27^. In frozen and quinone-blocked RC of the purple bacterium *Rhodobacter (R.) sphaeroides* wild-type (WT), which is the best studied photosystem^28,29^, the SCRP is formed by fast electron transfer from the electron donor, the so-called special pair, composed by the two bacteriochlorophyll (BChl) cofactors P_L_ and P_M_, to the primary electron acceptor, a bacteriopheophytin Φ. That system has been studied so far in a field-range between 1.4 and 17.6 T showing a strong field-dependence^6,19^. After reaching a maximum at about 5 T, the strength of the effect decays with increased magnetic field^6^. Towards lower fields, hyperpolarization and spectral dispersion decrease and hardware limits do not allow for proton decoupling, causing a broad spectral “hump” without chance of more detailed analysis^19^. Therefore, a complete curve for the high-field dependence of the solid-state photo-CIDNP effect has not yet been established.

Here we introduce a field-cycling technique called photo-CIDNP shuttle-MAS NMR. Our new setup allows to explore more electron-transfer systems at plenty of other fields, and it allows to explore the field ranges of the occurrence of the effect. Here, as a first step, we present the field-dependence of the solid-state photo-CIDNP effect in selective isotopically labelled RCs of *R*. *sphaeroides* WT below 2 T using an aerodynamic shuttling technique measuring at 9.4 T under MAS and at low temperature.

## Results and Discussion

### Field-cycling MAS NMR setup

**Figure 1:**
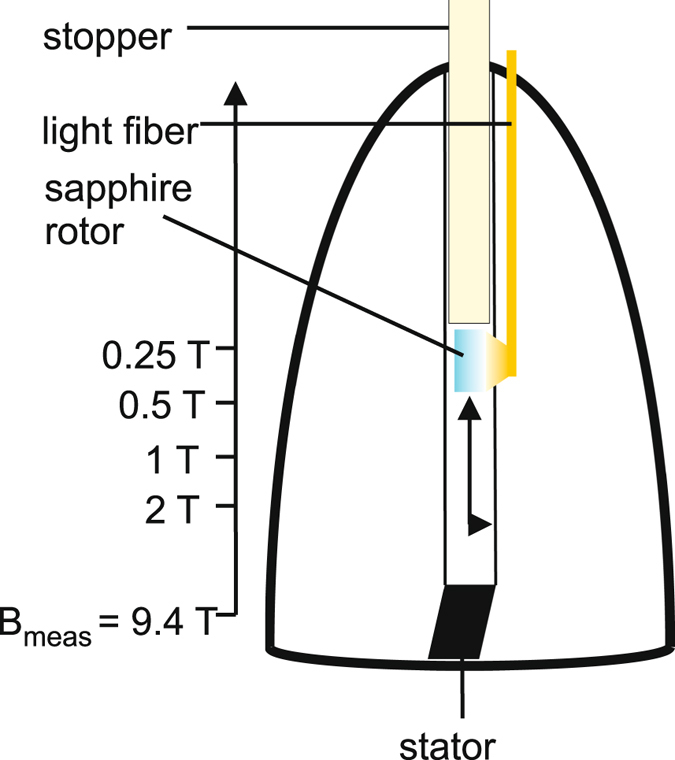
Schematic representation of the aerodynamic field-cycling setup using a wide-bore NMR magnet system. At the beginning of a shuttle cycle, the MAS rotor is located in the stator at the magnetic field B_meas_. The rotor is lifted into the stray field of the magnet by a continuous gas flow and carried to the desired position where the illumination takes place. Afterwards, the gas flow is stopped allowing the rotor to return into the high-field position where the NMR measurement takes place after a MAS spinning frequency of 8 kHz has been reached.

**Figure 2:**
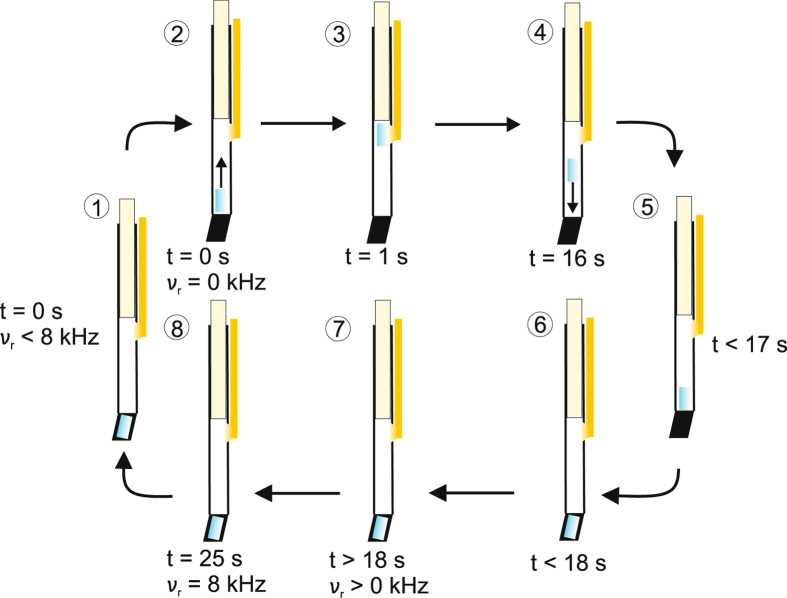
Schematic representation of the time schedule of the aerodynamic field-cycling setup using a wide-bore NMR magnet system. At the beginning of a shuttle cycle, the MAS rotor is located in the stator at the magnetic field B_meas_ and the spinning is stopped (1). It is lifted into the stray field of the magnet by a continuous gas flow (2) and carried to the desired position where the illumination takes place (3). Afterwards, the gas flow is stopped (4) allowing the rotor to return into the high-field position (5) where it is inserted into the stator (6) and accelerated (7). The NMR measurement takes place after a MAS spinning frequency of 8 kHz has been reached (8).

Figure 1 shows the field-cycling MAS NMR setup: the MAS rotor, initially located in the stator, i.e., within the homogenous field (B_meas_) in the interior of the MAS NMR probe, is transferred into the stray field of the magnet by pressurized gas flow and halted at the desired position by a mechanical stopper. At this position, the rotor is illuminated with intense white light. The inhomogeneity in the stray field of the magnet at the illumination position over the sample volume was found to be 240 G/mm at the 2 T position, 100 G/mm at the 1 T position, 45 G/mm at the 0.5 T position and 15 G/mm at the 0.25 T position. Such variations are not expected to affect the sign of the light-induced signal^19^. After illumination of the sample in the stray field, the gas flow is stopped, allowing the rotor to return into its high-field position (B_meas_) where the measurement takes place after a MAS spinning speed of 8 kHz is reached. A full shuttle-cycle including illumination lasts 25 s independent of the illumination position. All timings can be freely adjusted and are controlled by a simple program implemented in the software used for the measurements. For a graphical presentation of the shuttle cycle, see Fig. 2.

### Field-cycling photo-CIDNP MAS NMR experiments

**Figure 3:**
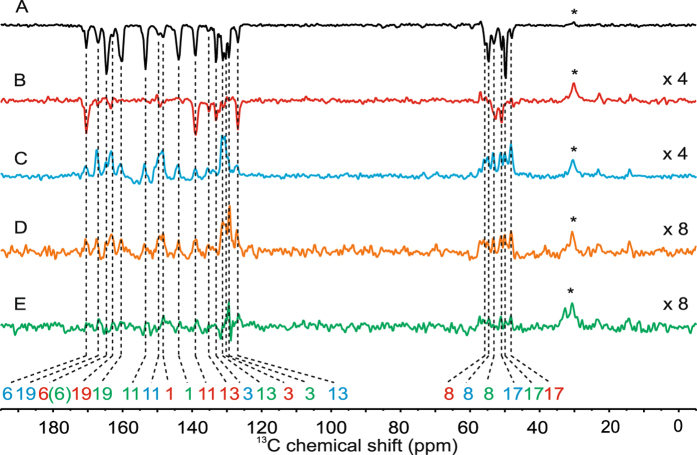
^13^C photo-CIDNP MAS NMR spectra of selectively ^13^C labeled bacterial RCs of *R*. *sphaeroides* WT measured at 9.4 T under 8 kHz MAS at a temperature of 250 K under continuous illumination (**A**) and after an illumination period of 15 s at 2.0 T (**B**), 1.0 T (**C**), 0.5 T (**D**) and 0.25 T (**E**) (see text for details). The color of the numbering refers to the assignment to three cofactors forming the spin-correlated radical pair: Green, red, and blue refer to the two bacteriochlorophyll *a* molecules of the donor (P_L_, P_M_) and the acceptor Φ, respectively. Cofactors are isotope labeled by feeding bacterial with 4-^13^C-δ-Aminolevulinic acid (4-ALA). For label pattern and nomenclature, see Fig. S2. The signal labelled with asterisk belongs to the 3300 methyl groups of the protein backbone and is not light-induced.

Figure 3 shows ^13^C MAS NMR spectra of a selectively ^13^C labeled RC of *R*. *sphaeroides* WT that were acquired after 15 s of illumination at a magnetic field of 2 T, 1 T, 0.5 T as well as 0.25 T (spectra B-E) in comparison to the spectrum acquired under continuous illumination at 9.4 T (spectrum A). The illumination period of 15 s has been chosen to ensure a full buildup of nuclear hyperpolarization due to the solid-state photo-CIDNP effect. The buildup has been shown to be faster than the *T*_1_ relaxation time of ^13^C nuclei of the cofactors^30^ which is about 20 s^31^. The *T*_1_ value is therefore also sufficiently long to survive the 9 s of sample transfer to the stator and reaching the MAS frequency of 8 kHz. The spectral dispersion of the stray-field induced spectra is comparable to the one obtained under stationary conditions at the same number of scans, proving the suitability of the shuttle-system for ^13^C photo-CIDNP MAS NMR. The lower signal-to-noise ratio is caused by relaxation during time before the measurement required for obtaining stable MAS. Due to sample cooling in the transfer tube, the sample does not melt during the illumination, and the measurements take place at a defined and stable temperature as indicated by the ^1^H wobble curve and the sample temperature after a few cycles. The lack of MAS during the illumination time does not interfere with the generation of the solid-state photo-CIDNP effect acting in the ns range since any interactions that would be averaged out by MAS (dipole-dipole interactions and chemical-shift anisotropy) act in the range of milliseconds. Spin diffusion, on the other hand, is expected to be enhanced accelerating the exchange of polarization among isotope labelled carbon positions on the cofactors^32^.

### Magnetic field-dependence of the solid-state photo-CIDNP effect

The solid-state photo-CIDNP effect in the RCs of *R*. *sphaeroides* WT, which has been demonstrated previously by static field experiments in the range between 1.4 and 17.6 T to be emissive^6,19,33–35^, also shows entirely emissive signal intensity at 2 T (Spectrum B in Fig. 3). Remarkably, all light-induced signals change their sign from emissive to enhanced absorptive in between 1.0 and 2.0 T (Spectrum C in Fig. 3). Since static experiments at 1.4 T, although conducted on a sample at natural abundance, show also emissive signals^19^, the sign change appears to occur between 1.0 and 1.4 T. At lower magnetic fields, the effect stays enhanced absorptive and decreases in magnitude (Spectra D and E in Fig. 3). Hence, our field-cycling data confirm the theoretical prediction of the existence of a high-field matching window for the occurrence of the effect^16,20^ which is now experimentally determined to occur between about 0.2 and 20 T having a maximum at about 5 T^20^.

The signals are assigned to particular carbons of the cofactors forming the SCRP, i.e., the two donor bacteriochlorophyll cofactors P_L_ and P_M_ as well as the bacteriopheophytin Φ (see Table S1). At 2 T, most of the signals disappear, while the signals of carbons 3, 6, 13, 17 and 19 of P_L_, 11, 13 and 19 of P_M_ and 8 and 13 of Φ stay emissive (spectrum B in Fig. 3). At 1 T all the signals re-appear as absorptive signals (spectrum B in Fig. 3) and decay at lower fields (spectrum D and E in Fig. 3). The origin of the sign change between 1.0 and 1.4 T remains unclear (see below). Apparently, the change of sign occurs in both the donor and the acceptor molecule^6^.

**Figure 4:**
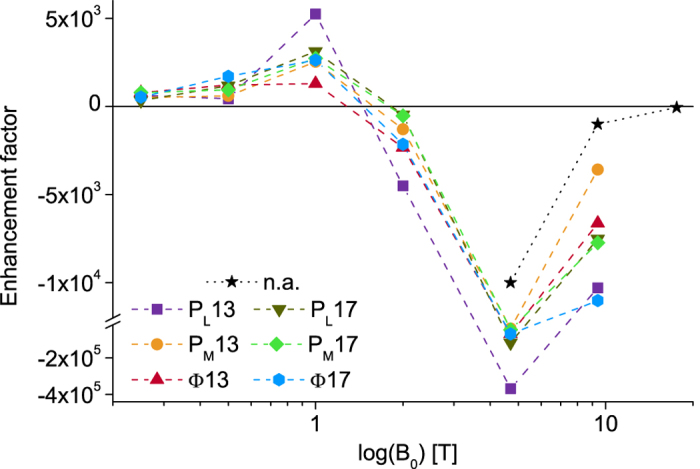
Magnetic field dependence of the photo-CIDNP enhancement factor for selected carbon positions in the selectively 4-ALA labeled bacterial RCs of *R*. *sphaeroides* WT in comparison with the natural abundance (n.a.) case observed in the unlabelled sample^6^.

Figure 4 shows the magnetic field dependence of the solid-state photo-CIDNP effect for an aliphatic (C-17) and an aromatic carbon position (C-13) in the special pair (P_L_ and P_M_) as well as in the bacteriopheophytin Φ. All signals show a similar field dependence and change sign between 1.0 and 2.0 T. Carbon C-13 in the bacteriochlorophyll *a* molecule P_L_ shows the strongest absolute enhancement of 370.000 at 4.7 T relative to the 3300 methyl groups at around 30 ppm (see asterisk in Fig. 3). The other positions show an absolute enhancement between 300 and 120.000. To the best of our knowledge, this exceeds the strongest signal enhancement for any macromolecule reported so far by at least an order of magnitude.

### Sign-field-relationship of the solid-state photo-CIDNP effect

For the unexpected change of sign, several reasons can be considered: (i) An additional source of polarization, namely the differential relaxation (DR) might start to contribute^16,19,20^. The DR mechanism is field-dependent and indeed has been demonstrated to be more pronounced at low magnetic fields^19^. On the other hand, it was shown that the effect of DR does not contribute to the photo-CIDNP-induced signal in the RCs of *R*. *sphaeroides* WT since the molecular triplet state is rapidly quenched by nearby carotenoids^19^. Furthermore, the DR mechanism enhances only the signals coming from the donor molecule^19^ which is in contrast to the observations of the present work (see Table S1). (ii) Similarly, a field-dependent loss factor, which causes nuclear polarization from the triplet branch to disappear and which was observed in the RCs of *R*. *sphaeroides* R26, requires long-lived donor triplet states^30^. (iii) The ratio between TSM and DD depends on the magnetic field. Nonetheless, preliminary simulations based on TSM and DD show no change of sign over the entire magnetic field range (see Fig. S4). Since lifetime of the radical pair is also field-dependent^36,37^ that might lead to a stronger DD contribution and therefore change the sign of the signal. (iv) An imbalance of both contributions might also be induced due to the anisotropy of the hfi. Since the matching conditions for TSM ($2|\Delta \Omega |=2|{\omega }_{I}|=|A|$) and the DD mechanism ($2|{\omega }_{I}|=|A|$) (Fig. S1; ΔΩ = difference in electron Zeeman frequencies; ω_I_= nuclear Zeeman frequency) are different but rely on the pseudo-secular part of the hyperfine interaction A, due to the anisotropy of the hfi, the matching conditions will only be fulfilled for some molecular orientations. If only the DD matching condition is fulfilled, then enhanced absorptive signals occur. Theoretical studies of orientational dependence of the solid-state photo-CIDNP effect as well as field-cycling experiments with different systems are under investigation to verify the findings. (v) Multi-spin effects might be responsible for the change of sign. In liquid-state photo-CIDNP, Kaptein’s sign rules have been violated when multiple spin labels were introduced^38–40^. Studies on a labeled LOV2-C450A domain showed that the number of spin-labels can change the sign of photo-CIDNP signals if solid-state photo-CIDNP mechanisms are considered^41^. Furthermore, addition of nuclear spins affect the coherent spin dynamics and therefore the built-up of polarization in bacterial RCs^42^. Preliminary calculations show no influence of multiple spins^15^, however the theoretical background for multiple spin labels in solid-state photo-CIDNP is still limited^16^. For further insight, whether multiple spin-labels cause a change of sign, experiments on the RCs of *R*. *sphaeroides* WT with a different number of ^13^C labels are presently on the way.

In any case, independent of details of the theory, our new technique allows for determination of magnetic field windows. The field-window of the occurrence of the effect appears to be significantly broader than that in the flavin protein phototropin LOV1-C57S^7^. The present observation underlines a concept proposed by Closs^43^ that the field-width of the enhancement window is inversely correlated to the lifetime of the SCRP. It is the physical perfection of the photosynthetic primary reaction, which does not allow for reaction branching, which allows for the extremely fast electron transfer, and therefore, for the broad field window, too. This physical basis allowing for perfect biological function (which remains in the quinone depleted sample) correlates with the solid-state photo-CIDNP effect even if the biological function is disabled. Furthermore, the extremely broad enhancement window explains the frequent observation of the effect in photosynthetic systems, while non-photosynthetic systems, being less efficient and having less broad field-windows, hardly show the effect.

The experimental observation of the low-field wing of the enhancement window of the solid-state photo-CIDNP effect demonstrates the applicability of the shuttle MAS NMR system. This device also might help to demonstrate the effect in more and non-photosynthetic electron-transfer systems opening a new avenue for the induction of hyperpolarization simply by sample illumination. Due to its easy implementation, it delivers a base for field-cycled solid-state dynamic nuclear polarization^44^ (DNP) and optical nuclear polarization^45^ (ONP) experiments under MAS, which so far were just done under static conditions^5,9,13,46^.

## Materials and Methods

### Sample preparation

The sample was prepared as given in ref.^47^. The isotope pattern obtained by labelling with 4- ^13^C-δ-Aminolevulinic acid (4-ALA) is shown in Figure S2.

### NMR Experiments

All NMR experiments were performed at 9.4 T with an AVANCE III NMR spectrometer equipped with a 4-mm double resonance MAS probe (Bruker, Karlsruhe, Germany). Approximately 5 mg of 4-ALA labeled purified RCs of *R. sphaeroides* (corresponding to ca. 70 μL at a concentration of 0.8 mM) were loaded into a transparent 4-mm sapphire rotor and inserted into the MAS probe. Freezing the sample for the solid-state NMR experiments was achieved by cooling the solution at 250 K in the magnet for one hour before starting the measurement. During this time the rotor was kept spinning at low frequency (800 Hz) to ensure a homogeneous sample distribution against the rotor wall. In case of field-cycling, it was checked by observation of the wobble curve that the sample is still frozen after a full shuttle cycle.

The solid-state spectra were collected at a spinning frequency of 8000 Hz with a single 90° pulse with an rf-field strength of 80 kHz. The FID was collected under SPINAL64 decoupling^48^ with an rf-field strength of 100 kHz. 64 transients were summed up for each spectrum.

Illumination of the sample was achieved by a 1000−W Xe arc lamp with collimation optics, a liquid filter and glass filters, a focusing element and a light fiber^31^. The light fiber was either mounted in the stator to illuminate the sample in the high field for steady-state experiments or mounted at the shuttle to ensure illumination at the desired position in the stray field.

## Electronic supplementary material

Supporting Information
