# Supplementary material for: Field-cycling NMR with high-resolution detection under magic-angle spinning: determination of field-window for nuclear hyperpolarization in a photosynthetic reaction center
Source: Sci Rep. 2017 Sep 21;7:12111. doi: 10.1038/s41598-017-10413-y (PMC5608766; doi:10.1038/s41598-017-10413-y)
Supplement: Supplementary file 1 — Supporting Information [file 41598_2017_10413_MOESM1_ESM.pdf]

Supporting Information

Field-cycling NMR with high-resolution detection under magic-angle spinning: determination of field-window for nuclear hyperpolarization in a photosynthetic reaction center

Daniel Gräsing[†], Pavlo Bielytskyi[†], Isaac F. Céspedes-Camacho[†,‡], A. Alia[ǂ], Thorsten Marquardsen[§], Frank Engelke[§], and Jörg Matysik[†,1]

† Institut für Analytische Chemie, Universität Leipzig, Linnéstraße 3, D-04103 Leipzig, Germany

‡ Escuela de Química, Tecnológico de Costa Rica, Sede Central, 30101 Cartago, Costa Rica

ǂ Institut für Medizinische Physik und Biophysik, Universität Leipzig, Härtelstr. 16-18, D-04107 Leipzig, Germany

§ Bruker BioSpin GmbH, Silberstreifen 4, D-76287 Rheinstetten, Germany

Photo-cycle describing the origin of the solid-state photo-CIDNP effect

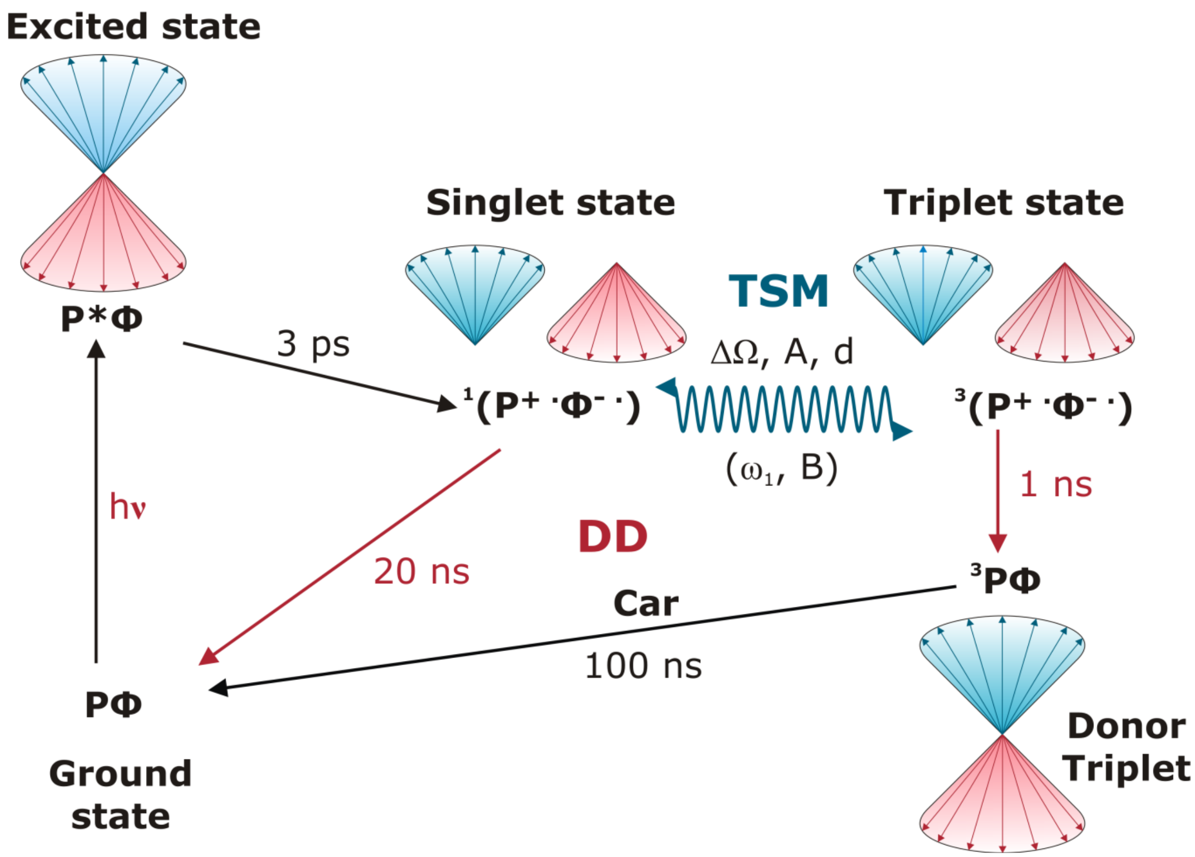

Figure S1: Kinetics and spin dynamics during the primary electron transport during the photo-cycle in the RCs of *R. sphaeroides* WT. After photo-excitation of the donor (P), an electron is transferred from to the acceptor (**Φ**), creating a spin-correlated radical pair (SCRP). While singlet-triplet interconversion, the SCRP evolves under the difference in electron Zeeman frequencies ($\Delta\Omega$), the nuclear Zeeman frequency (ω_1), the secular (A) as well as the pseudo-secular (B) part of the electron-nuclear hyperfine interaction (hfi) and the electron-electron dipolar interaction (d) (4). This evolution leads to the creation of nuclear hyperpolarization due to the three-spin mixing (TSM) mechanism (2, 4). Additionally, due to the different reaction rates of the radical-pair singlet state to the electronic ground state and the radical-pair triplet state to the molecular donor triplet state, the differential decay (DD) mechanism occurs based on the anisotropic components of the electron-nuclear hyperfine interaction (4, 5).

Signal assignment

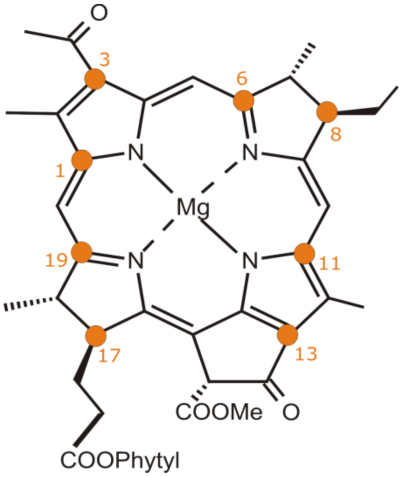

Figure S2: Bacteriochlorophyll *a* according to IUPAC nomenclature. Orange circles mark the positions of the ^{13}C-labels upon selective isotope labelling with 4-^{13}C-δ-Aminolevulinic acid (4-ALA). The label pattern is the same for the two donor cofactors (P_M and P_L) and the acceptor bacteriopheophytin *a* (**Φ**).

Table S1: Strength of the CIDNP signal (s = strong, m = medium, w = weak, 0 = no signal) of each cofactors at different magnetic fields. The chemical shift assignment is based on ref. 36.

Cofactor	Atom number	Chemical shift [ppm]	Magnetic field [T]	Signal	Strength of the CIDNP
P_L	1	143.6	9.4 T	e	s
			2 T	0	0
			1 T	a	m
			0.5 T	a	m
			0.25 T	0	0
	3	129.1	9.4 T	e	s
			2 T	0	0
			1 T	a	m
			0.5 T	a	s
			0.25 T	0***	0***
	6**	164.5	9.4 T	e	s
			2 T	0	0
			1 T	a	m
			0.5 T	a	m
			0.25 T	0	0
	8	53.0	9.4 T	e	m
			2 T	e	m
			1 T	a	m
			0.5 T	a	w
			0.25 T	0	0
	11	153.2	9.4 T	e	s
			2 T	0	0

			1 T	a	m
			0.5 T	a	w
			0.25 T	0	0
	13	131.0	9.4 T	e	s
			2 T	e	w
			1 T	a	s
			0.5 T	a	s
			0.25 T	0	0
	17	49.7	9.4 T	e	s
			2 T	0	0
			1 T	a	s
			0.5 T	a	m
			0.25 T	0	0
	19	160.0	9.4 T	e	s
			2 T	0	0
			1 T	a	w
			0.5 T	a	w
			0.25 T	0	0
P_M	1	148.2	9.4 T	e	w
			2 T	0	0
			1 T	a	s
			0.5 T	a	m
			0.25 T	a	w
	3	130.2	9.4 T	e	s
			2 T	0	0
			1 T	a	s
			0.5 T	a	m
			0.25 T	0	0
	6**	164.5	9.4 T	e	s
			2 T	0	0
			1 T	a	m
			0.5 T	a	m
			0.25 T	0	0
	8	55.5	9.4 T	e	m
			2 T	0	0
			1 T	a	m
			0.5 T	a	m
			0.25 T	a	w
	11	138.9	9.4 T	e	s
			2 T	e	s
			1 T	a	w
			0.5 T	a	m

			0.25 T	0	w
	3	135.3	9.4 T	e	w
			2 T	e	m
			1 T	a	w
			0.5 T	a	w
			0.25 T	0	0
	17	47.9	9.4 T	e	w
			2 T	0	0
			1 T	a	s
			0.5 T	a	m
			0.25 T	a	w
	19	162.6	9.4 T	e	w
			2 T	e	w
			1 T	a	s
			0.5 T	a	m
			0.25 T	0	0
**Φ**	1*	-	9.4 T	-	-
			2 T	-	-
			1 T	-	-
			0.5 T	-	-
			0.25 T	-	-
	3	132.9	9.4 T	e	s
			2 T	e	m
			1 T	a	w
			0.5 T	0	0
			0.25 T	0	0
	6	170.3	9.4 T	e	m
			2 T	e	s
			1 T	a	w
			0.5 T	a	w
			0.25 T	0	0
	8	54.6	9.4 T	e	s
			2 T	0	0
			1 T	a	m
			0.5 T	a	m
			0.25 T	a	w
	11	149.5	9.4 T	e	w
			2 T	0	0
			1 T	a	s
			0.5 T	a	m
			0.25 T	0	0
	13	126.6	9.4 T	e	m
			2 T	e	s

			1 T	a	w
			0.5 T	a	m
			0.25 T	a	w
	17	50.8	9.4 T	e	m
			2 T	e	m
			1 T	a	s
			0.5 T	a	s
			0.25 T	a	w
	19	166.9	9.4 T	e	m
			2 T	e	w
			1 T	a	s
			0.5 T	a	w
			0.25 T	a	w

* no signal could be assigned; ** signal assignment was unclear, *** signal overlapped with the offset

SIMULATIONS

Simulations were carried out using a home written MATLAB script as described in earlier works.[18] Figure S3 shows the trend of the enhancement due to the solid-state photo-CIDNP effect with the magnetic field for two selected nuclei in the donor (P_M) and the acceptor (**Φ**).

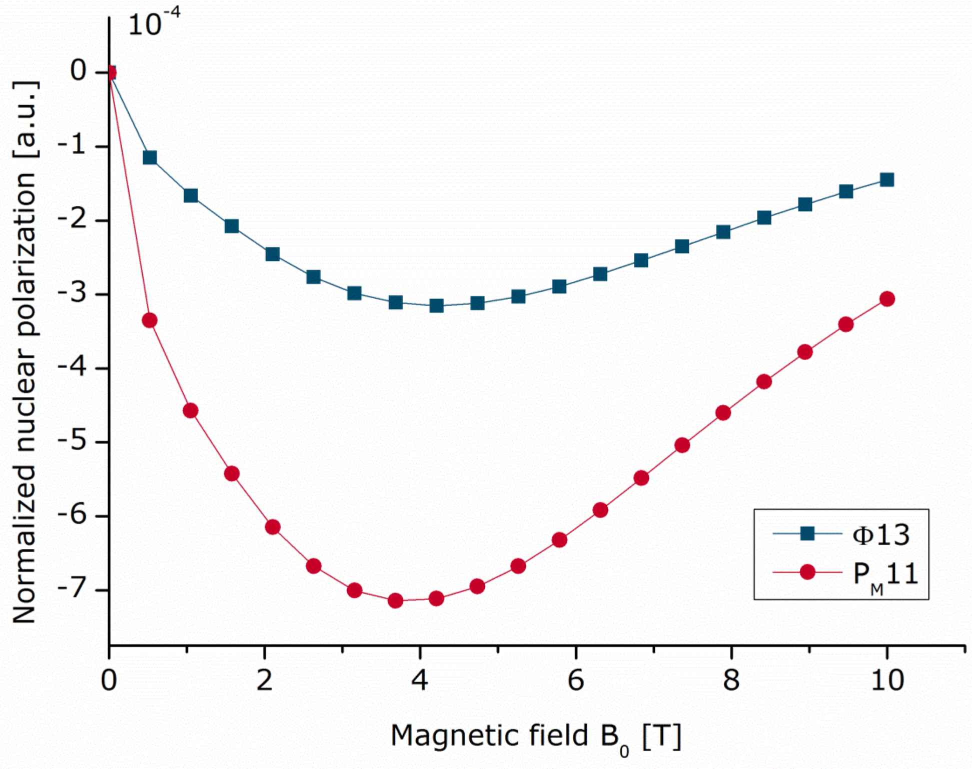

Figure S3: Simulated trend for the enhancement due to the solid-state photo-CIDNP effect as a function of the magnetic field for two selected nuclei from the donor (P_M) and the acceptor (**Φ**) cofactors. For nomenclature, see Fig. S2.
